# Analysis of canopy phenology in man-made forests using near-earth remote sensing

**DOI:** 10.1186/s13007-021-00803-9

**Published:** 2021-10-12

**Authors:** Peng Guan, Yili Zheng, Guannan Lei

**Affiliations:** 1 School of Engineering, Beijing Forestry University, Beijing, China; 2 Beijing Laboratory of Urban and Rural Ecological Environment, Beijing Municipal Education Commission, Beijing, China

**Keywords:** Color index, Forecast, Forest phenology, LSTM model, Near-earth remote sensing

## Abstract

**Background:**

Forest canopies are highly sensitive to their growth, health, and climate change. The study aims to obtain time sequence images in mix foresters using a near-earth remote sensing method to track the seasonal variation in the color index and select the optimal color index. Three different regions of interest (RIOs) were defined and six color indexes (GRVI, HUE, GGR, RCC, GCC, and GEI) were calculated to analyze the microenvironment difference. The key phenological phase was identified using the double logistic model and the derivative method, and the phenology forecast of color indexes was performed based on the long short-term memory (LSTM) model.

**Results:**

The results showed that the same color index in different RIOs and different color indexes in the same RIO present a slight difference in the days of growth and the days corresponding to the peak value, exhibiting different phenological phases; the mean squared error (MSE), root mean squared error (RMSE), mean absolute error (MAE), and mean absolute percentage error (MAPE) of the LSTM model was 0.0016, 0.0405, 0.0334, and 12.55%, respectively, indicating that this model has a good forecast effect.

**Conclusions:**

In different areas of the same forest, differences in the micro-ecological environment in the canopies were prevalent, with their internal growth mechanism being affected by different cultivation ways and the external environment. Besides, the optimal color index also varies with species in phenological response, that is, different color indexes are used for different forests. With the data of color indexes as the training set and forecast set, the feasibility of the LSTM model in phenology forecast is verified.

## Background

The the past half century saw a tendecy towards sustainable management of natural and artificial forests in many countries around the world. The greatest achievements in the field of forest policy took place in the European Union and the United States [1], where many institutions aim at promoting the effective use of forests. Countries in Europe have been making joint efforts to protect European forests since 1990. Those include the consolidation of funds for forest management, a fight against illegal logging, and awareness campaigns aimed at drawing attention to the social aspects of forestry and promoting the role of forests in green economies [2, 3]. The U.S. Forest Service, a federal agency in natural resource conservation, provides guidance on national forests, grassland and aquatic ecosystems to maintain the health, diversity, and productivity of forests and grasslands to meet th needs of present and future generations [4]. Reforestation in the United States began with Henry Hardtner in 1912, who bought out the deforested areas for replanting, assuming that commercial crops still can be grown there after 60 years of recovery [5]. As an initiator of forest conservation and reforestation, Hardter invested heavily in forest research and involved many other scientists. One of them was W.R. Mattoon, who drew up a preliminary plan for forest thinning experiments and wildfire protection in 1913. Henry Hardtner became a Father of Southern Forestry in 1917, a head of the Natural Resources Conservation Commission, and a successful legislator in Louisiana. Hardtner was one of the first people to take advantage of reforestation and sustainable yield measurement techniques and establish ties with the U.S. Forest Service and Yale’s Forestry School [6].

In most cases, institutions for forest conservation seek to facilitate sustainable development and solve problems associated with natural forest ecosystems. Meantime, the artificial forests also have the potential to become an important part of sustainable development in industrial areas, where anthropogenic impacts have a strong effect on the environment [7]. Man-made forests are encountering various adverse factors such as single tree species, insufficient soil fertility, weak ability to resist plant diseases and insect pests as well as water and soil loss caused by human interference and unbalance of nutrient income and expense in the forest ecosystem caused by emigration of a large amount of biomass, resulting in a gradually degraded forest environment. Forest canopy can regulate photosynthesis and its related ecosystem processes and is highly sensitive to its growth and health as well as climate change. Therefore, monitoring the phenological dynamics of forest canopies is of great significance to mastering forest growth status and predicting climate change.

Much labor is required to acquire the earth’s phenology records of forests. To this end, satellite remote sensing technology was developed and has gradually become a mainstream method to monitor canopy phenology, which can achieve phenological observation on regional and global scales. However, monitoring plant phenology using satellites has always been restricted because its spatial scale and observation time are quite different from the artificial observation data on the earth, and it is affected by external factors like clouds and atmosphere [8]. Also, this method has insufficient spatial resolution and low revisit frequency defect [9–12], thus making it more difficult to obtain precise, abundant, and valuable phenological phases of vegetation. This problem is even more serious in man-made forests in urban areas. There are many high-rise buildings and scattered green plants in the city, so it is difficult to use publicly available satellite data for identification [13–15]. In contrast, near-earth remote sensing technology monitors the phenological dynamics of forest vegetation with a camera mounted on a tower with a height of not greater than 40 m, which has the advantages of high temporal resolution and moderate spatial scale [16]. Since near-earth remote sensing has advantage like automatic high-frequency measurement, high-quality images, and low cost, digital repeat photography has become an important means to observe forest phenology in situ [17–21], which makes up the gap between the phenological variables estimated by satellite and the field observation data [18]. Near-earth remote sensing is a technology that continuously obtains images of the canopy in a season and extracts the phenology and growth status by using the red, green, and blue (RGB) color information contained in these images [14, 19, 20].

At present, most camera-based phenology studies only focus on a single region of interest (ROI) in the image to track vegetation phenology and consider this region as a reference for the average behavior of the entire ecosystem [19–21]. Few studies have used multiple ROIs to evaluate phenological differences between different species or even different individuals in the same canopy image [22]. Besides, there is no unified conclusion on the optimal color index for monitoring the phenological change of canopy [23]. So far, only the random forest regression model is used to predict the yield of crop vegetation [24] and transpiration of leaf area [25], and forest fires are predicted based on the diagonal recurrent neural network (DRNN), but no predictions have been made in phenological studies.

From the aforesaid, three problems were discussed in this paper: (1) the growth status of the entire forest will not be well reflected if only a single ROI is studied, and there may be different micro-ecological environments in the forest canopy; (2) although there are differences among different color indexes, they may all reflect the forest phenology, and the optimal one depends on tree species; (3) based on years of experience, people who cultivate man-made forests will provide different amounts of forest nutrients, such as irrigation water, which will trigger different changes in their internal physiological mechanism. In this case, the escalation of pests and diseases in the microenvironment around the forests is greatly nurtured due to the waste of nutrients and water loss. The can be attributed to the inability to accurately identify much-needed nutrients in different locations. In this study, near-earth remote sensing monitoring method is used to extract image data from the experimental base in Louisiana in the southern part of the United States for purposes as follows: (1) to observe the seasonal variation characteristics of the color index at the same position and different positions according to camera images, and understand the canopy growth at different positions and the differences between different color indexes; (2) to select the optimal color index and determine the key phenological phase based on the double logistic model and the derivative method, and realize phenology forecast based on the long short term memory (LSTM) model. This is conducive to understanding the growth at different positions, achieving scientific and precise irrigation, the addition of nutrients, and other factors affecting healthy growth. Meanwhile, it provides a theoretical basis for more scientific cultivation.

## Methods

### Overview of the study area

**Fig. 1:**
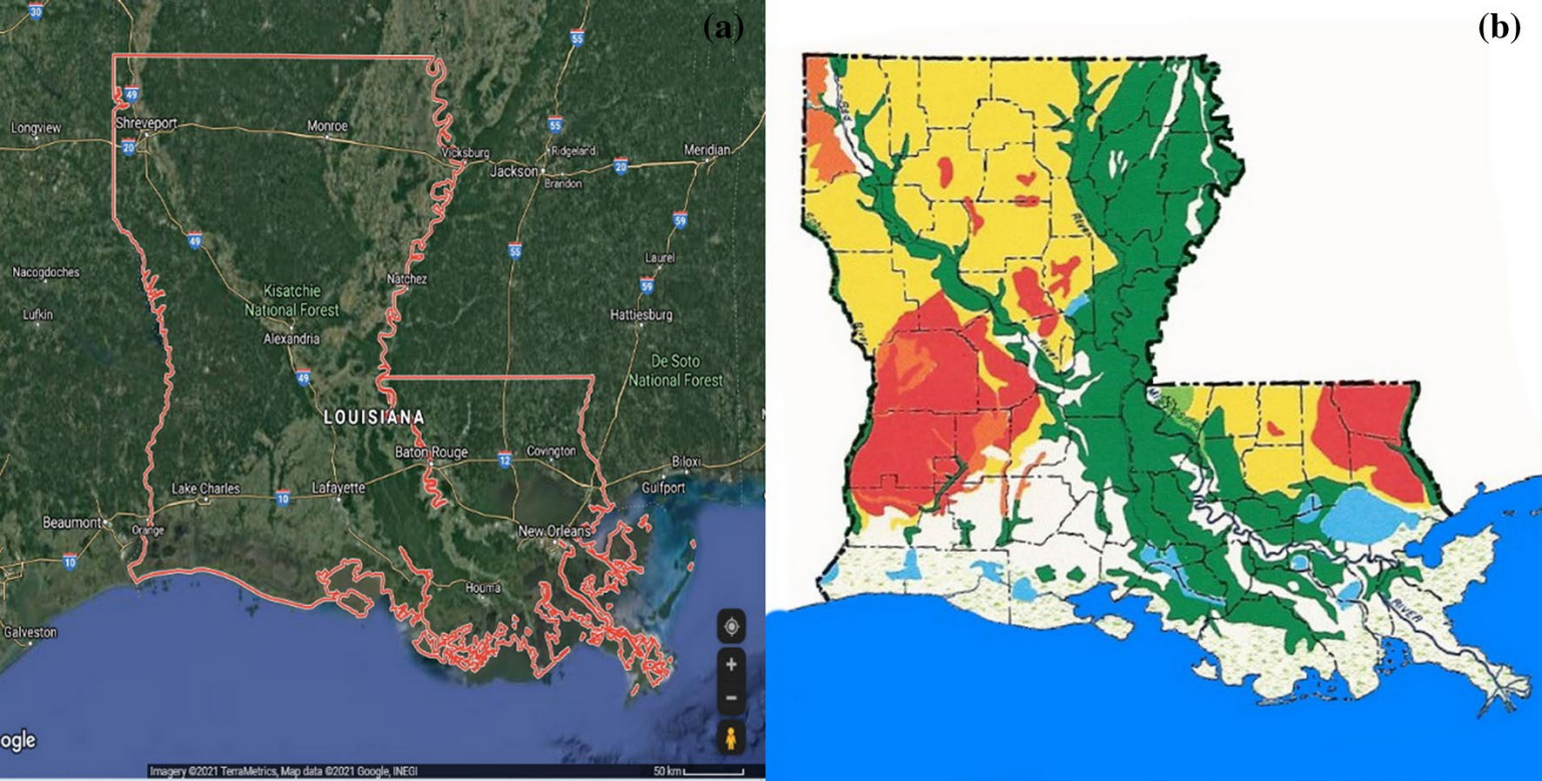
(**a**) Geographical area of the study. (**b**) The forest types of Louisiana: orange - OakPine; yellow - loblolly-shortleaf Pine; green - OakGum-Cypress; red - longleaf-slash Pine; white areas - nontyped

This study was carried out in Louisiana (N32.45°, W91.97°), which is located along the Gulf of Mexico, bordering Arkansas in the north, Texas in the west, Mississippi in the east, and the Gulf of Mexico in the south (see Fig. 1a). It is one of the main producing areas of wood, paper products, and wooden boards in America. The forest area is 590 hm^2^, accounting for 47% of the land area of the state. This state is in the subtropical moist monsoon climate zone, and the average growth period is 220–320 days.

Forests cover about 50% of Louisiana's land area and make up more than 15 million acres, of which more than 1 million acres are artificial stands [26]. Private non-industrial landowners own 81% of the state forest land, while forestry enterprises and the government own 10 and 9%, respectively. Landowners in Louisiana plant more than 130 million seedlings each year to restore the forest area. Figure 1b shows the main types of forests in the state. The dominant forest types appear to be *coniferous forests*, such as loblolly/shortleaf pine (yellow), longleaf/slash pine (red), and mixed oak/pine (orange area) forests and *deciduous veegetation* like the Oak-Gum-Cypress (green) [27].

There are several differences between natural and man-made forests. Natural ecosystems have a diverse range of plant species and result from a spontaneous natural response. Man-made forests are limited and require human intervention. Natural forests are characterized by genetic diversity, complex food chains, efficient nutrient cycling, and ecological continuity. Artificial habitats are less resistant to climatic and biogenic changes and may perish without the supply of artificial fertilizers. The food chains in artificial forests are simple and often incomplete because other species are killed as pests and weeds.

### Data acquisition

Oak was selected as a monitoring object in mix forest of Caldwell (Louisiana) which had 52.7 kha of natural and man-made forests. A commercial web camera (DS-2DE7223IW-A/C) was used as the near-earth remote sensing device to obtain the time sequence images. The camera was mounted at a height of 25 m above the ground at the upper end of the flux tower in the center of the forest (facing northeast). The image sensor was 1/2.8 progressive scan CMOS, the infrared effective range was 150 m, and the camera operation mode was set to "automatic mode" for exposure and white balance adjustment. Since the mounting position is high and easily affected by external environments such as wind and rain, and the inevitable movement may lead to changes in the field of view, azimuth calibration and image registration are required. First, the camera was turned to 0, and then returned to the original azimuth after 1–2 turns, then azimuth calibration was completed. Next, image sequences were registered based on the area matching method, and the time display format was adjusted to facilitate the formation of the time sequence. To minimize the possible effects of different solar angles, the range of shooting time was from 7:05 A.M. to 17:05 P.M. every day with an interval of 1 h, and the images were saved in an uncompressed format as JPEG with a resolution of 2560 × 1440 pixels. The original images were transmitted through the wireless network and stored in the server according to the shooting time and place for subsequent arrangement. This study involvs a total of 4260 images made in November 01, 2017, to December 31, 2018.

### Image processing

#### 1. Calculation of color indexes

The image quality is sometimes adversely affected by variable light conditions, rain, snow, fog, or frost on the camera window. Therefore, before image analysis, no archive images are selectively edited or manually enhanced, and the time sequence generated by image analysis is not smoothed or filtered, to maintain objectivity to the greatest extent.

In this study, three regions of interest (ROIs, Fig. 1) were selected in each image, and many images on an hourly interval were processed in pyCharm compiler according to the algorithm "image processing tool" designed by python language, and the brightness values of R, G, and B of ROIs of each frame were extracted from the images. In order to reduce the color balance changes affected by fog and shadow, the mean value method was used to average the RGR brightness values of all images every day in a period of 1 d, and the time sequence plot of six color indexes, namely, GRVI, HUE, GGR, RCC, GCC, and GEI, were calculated and obtained to quantify the dynamics of vegetation canopy (Table 1).

**Table 1:** Equations of color indices measured

Color index	Equation	References
Ratio greenness index	$GGR=G/R$	[19]
Green chromatic coordinate	$GCC=G/(R+G+B)$	[20]
Green excess index	$GEI=2G-(R+B)$	[20]
Red chromatic coordinate	$RCC=R/(R+G+B)$	[21]
Green red vegetation index	$GRVI=(G-R)/(G+R)$	[22]
Hue	$HUE=(B-R)/({I}_{max}-{I}_{min}) x60+120$ $G={I}_{max}$ $HUE=(B-R)/({I}_{max}-{I}_{min}) x60+240$ $B={I}_{max}$ $HUE=(G-B)/({I}_{max}-{I}_{min}) x60+340$ $G<B$ $HUE=(G-B)/({I}_{max}-{I}_{min}) x60$ other	[23]

R, G, and B represented the brightness of red, green, and blue channel, respectively; ${\mathrm{I}}_{\mathrm{max}}$ and Imin represented the maximum and minimum of R, G and B, respectively

#### 2. Time extraction of vegetation growth characteristics

In this paper, the double logistic model (Eq. 1) and the curve-based derivative method (Eq. 2) were used to smooth and fit the seasonal variation curve of color indexes. The extreme point of the rate of change of curvature was taken as the phenological turning point of the community [28], corresponding to the start of the growing season (SOS) and the time point of exuberance [29], the start of corruption stage (COS) and the end of the growing season (EOS), respectively.

1 $$g\left(t\right) \,= \, \left(m-W\right)\left\{\frac{1}{1+\mathrm{exp}(-mDx\left(t-S\right))}+\frac{1}{1+\mathrm{exp}(-mAx\left(t-A\right))}\right\}-1+W$$

wherein: g(t) is the fitted value of color indexes; t is the day of the year (DOY); m is the minimum value and w is the maximum value in a year; parameters S and A represent the inflection points of start and end (SOS, EOS) of a year, respectively; mS and mA are the velocities at points S and A, respectively.

This curvature is related to the change of color indexes. The rate of change of curvature can explain the change speed and reflect each change node in the growth stage and the corruption stage. Based on the derivative formula of the curve as follows:

2 $$p = \left| {\frac{{g\left( t \right)^{{\prime \prime }} }}{{\left( {1 + g\left( t \right){^{\prime } {2} }} \right)^{{\frac{3}{2}}} }}} \right|$$

wherein: $g{(t)}^{^{\prime}}=\frac{dg(t)}{dt}$ // is the first-order derivative of $g(t)$; $g{(t)}^{"}=\frac{{d}^{2}g(t)}{{d}^{2}t}$ // is the second-order derivative of $g(t)$.

**Fig. 2:**
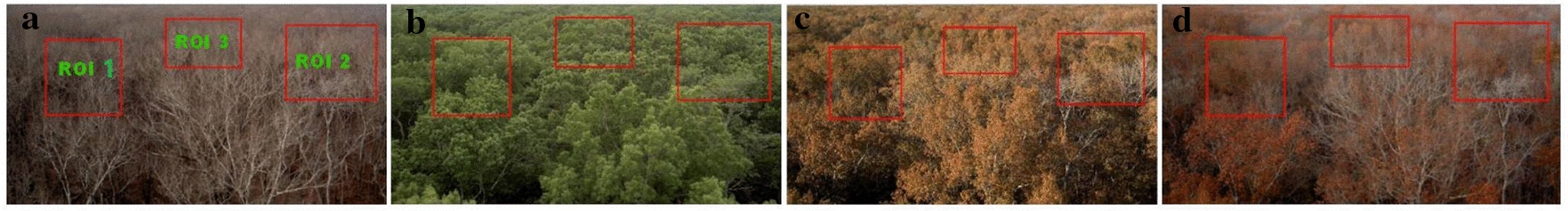
Region of interest within the image in an year : (**a**) spring; (**b**) summer; (**c**) autumn; (**d**) winter

Digital images obtained by cameras can be used to visually explain seasonal changes in vegetation: in winter and early spring, there were only bare trunks and other natural phenomena, when the vegetation was in the dormant period, and the color of the image was mainly that of the trunks (Fig. 2a); with the change of external environment such as temperature, vegetation began to grow (SOS), and the digital image dominated by the color of trunks was gradually replaced by scattered green. After the vegetation entered the exuberant growth period, green becomes the dominant tone of the image (Fig. 2b); with time, the leaves gradually turned yellow, the vegetation gradually entered the corruption stage, and the color of the image gradually changed from green to brownish-yellow (Fig. 2c); Until the end of the growing season (EOS) of vegetation, the digital image shows the color of trunks again (Fig. 2d).

### LSTM model

**Fig. 3:**
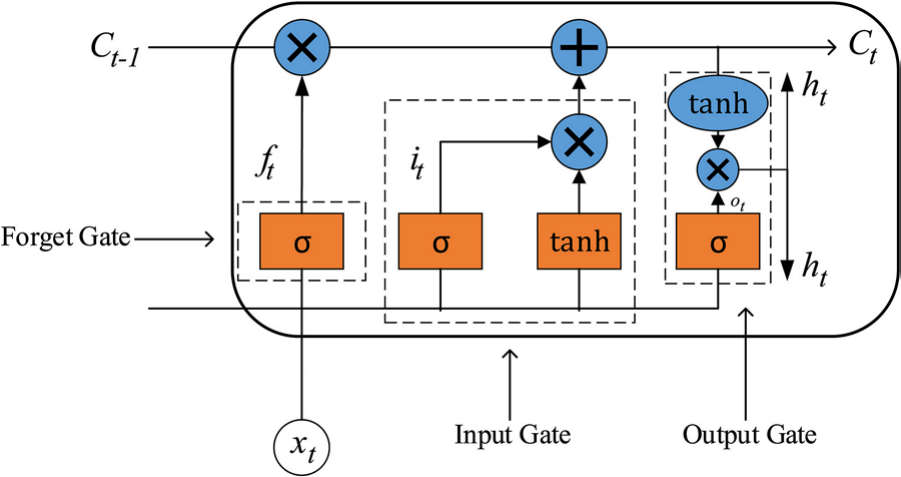
LSTM Structure of hidden layers

A recurrent neural network (RNN) is a kind of recursive neural network, which takes a sequence as input, recurs in the development direction of sequence and all nodes (recurrent units) are connected in a chain. RNN is also one of the deep neural network learning models, which can learn the long-term dependence. Long short term memory (LSTM) is a machine learning algorithm with recurrent neural network architecture, which is a special form of RNN. LSTM is essentially consistent with the principle of RNN, but the former is provided with three gate controls, namely, forgetting gate, in-gate, and out-gate. In other words, a memory block is used to replace the basic unit of conventional RNN to protect and control the unit state, and a "memory cell state" is introduced to store it for a long time (Fig. 3) [30, 31]. A phenological canopy color index prediction model based on LSTM is constructed according to the basic principle. In the prediction of phenological canopy color index based on the LSTM model, f^t^ is the control function of forgetting gate, which is denoted as

3 $${f}_{t}=\sigma ({W}_{f}\cdot \left[{h}_{t-1},{x}_{t}\right]+{b}_{f})$$

where ${W}_{f}$ is the weight matrix of the true value of the phenological canopy color index of forgetting gate; ${x}_{t}$ is the input at time $t$; ${h}_{t-1}$ is the predicted value of the phenological canopy color index at the previous time; ${b}_{f}$ is the deviation vector of forgetting gate; and $\sigma$ is the activation function Sigmoid.

${i}_{t}$ is the control function of in-gate, which is similar to ${f}_{t}$ and used to select the newly input phenological canopy color index data by changing the value of the weight matrix, thereby preventing the phenological canopy color index data with low correlation from being added to the memory cells. It is denoted as:

4 $${i}_{t}=\sigma ({W}_{i}\cdot \left[{h}_{t-1},{x}_{t}\right]+{b}_{i})$$

wherein: ${W}_{i}$ is the weight matrix of the true value of phenological observation color index of in-gate; ${h}_{t-1}$ is the state of hidden layer at the previous time; $\left[{h}_{t-1},{x}_{t}\right]$ is a long vector connected by two vectors; ${b}_{i}$ is the deviation vector of in-gate.

The phenological canopy color index vector at the current time is selected, converted and stored. The storage vector of memory cells at this time is to multiply the storage vector at the previous time and the newly input color index information vector at the current time with the control function vectors of forgetting gate and in-gate element by element, so as to realize the memory storage of phenological canopy color index information for a long time. ${C}_{t}$ is used to update the cell state, that is, to memorize the historical travel index with strong correlation. This variable adds new candidate variables by abandoning part of the memory at the previous time, thereby realizing continuous update and storage, which is denoted as:

5 $$\overline{{C}_{t}}=\mathrm{tanh}({W}_{c}\cdot \left[{h}_{t-1},{x}_{t}\right]+{b}_{c})$$

6 $${C}_{t}={f}_{t}\odot {C}_{t-1}+{i}_{t}\odot \overline{{C}_{t}}$$

wherein: $\odot$ is the symbol of vector element multiplication; $\mathrm{tanh}$ indicates that the value is scaled to the range of [−1,1] by tangent hyperbolic function. $\overline{{C}_{t}}$ is the tanh function conversion value of the actual value of the color index at the current time, which is taken as the color index vector at the current time; ${W}_{c}$ is the stored value weight matrix of phenological canopy color index at the current time; ${b}_{c}$ is the deviation vector at the current time.

Output control of the predicted value of the color index at the current time through the control function ${O}_{t}$ of out-gate is denoted as:

7 $${O}_{t}=\sigma ({W}_{O}\cdot \left[{h}_{t-1},{x}_{t}\right]+{b}_{O})$$

8 $${h}_{t}={O}_{t}\odot \mathrm{tanh}({C}_{t})$$

wherein: ${W}_{O}$ is the output weight matrix; ${b}_{O}$ is the deviation of out-gate.

A dataset consists of 4260 images collected over a period of 14 months. It was divided into a training set (70%), a testing set (20%) and a verification set (10%). The depth of the LSTM network was set to 2. The weights are initialized to the range of [−0.01, 0.01], and the distribution is uniform. The learning rate is set to 10^–5^ and decreases exponentially during training. The analysis involved assessing the accuracy of image recognition on an expanded set of 4260 frames. The trained model is evaluated using a verification set (which is 10% of the total data).

### Phenology forecast

A total of 14 monthly color index data instances from November 2017 to December 2018 were selected as the optimal color index. The selected data set was relatively complete and there was no missing value. Therefore, the calculation could be directly performed with the original data. After complete raw data was obtained, Jupyter Notebook was operated based on Python3.6 software. First, the required database was introduced to define the data and establish data sets. Next, the data was read and normalized. Finally, the model was constructed to divide the data sets into two types, i.e., the data of 10 months from November 2017 to August 2018 was used as the training set for the training model, and the data of 8–12 months in 2018 was used as the test set.

### Model evaluation

To accurately evaluate the forecast effect of the LSTM model, the mean squared error (MSE), the root mean squared error (RMSE), the mean absolute error (MAE), and the mean absolute percentage error (MAPE) were used as the basis to judge the forecast effect of LSTM model [29, 32, 33].

9 $$MSE=\frac{1}{N}\sum_{t-1}^{N}{({y}_{t}-\overline{{y}_{t}})}^{2}$$

10 $$RMSE=\sqrt{\frac{1}{N}\sum_{t-1}^{N}{({y}_{t}-\overline{{y}_{t}})}^{2}}$$

11 $$MAE=\frac{1}{N}\sum_{t=1}^{N}({y}_{t}-\overline{{y}_{t}})$$

12 $$MAPE=\frac{1}{N}\sum_{t=1}^{N}\left|\frac{{y}_{t}-\overline{{y}_{t}}}{{y}_{t}}\right|$$

where n is the total amount of data; ${y}_{t}$ is the true value; $\overline{{y}_{t}}$ is the predicted value.

## Results

### Comparison of the same color index in different ROIs

Six color indexes were extracted from different ROIs. The results showed that the green–red vegetation index (GRVI), the hue (HUE), the specific green index (GGR), the green chromatic coordinate (GCC), and the absolute green index (GEI) of the three ROIs could basically reflect the growth and corruption processes. The whole process corresponds to three stages of vegetation phenology: the first stage is the rapid growth period, where the corresponding GRVI, HUE, GGR, GCC, and GEI all show a trend of rapid growth; the second stage is the mature period, where color index remains relatively stable and shows slight fluctuation; the third stage is the corruption period, where the corresponding GRVI, HUE, GGR, GCC, and GEI drop to the lowest value, while the red chromatic coordinate (RCC) shows the opposite trend.

**Fig. 4:**
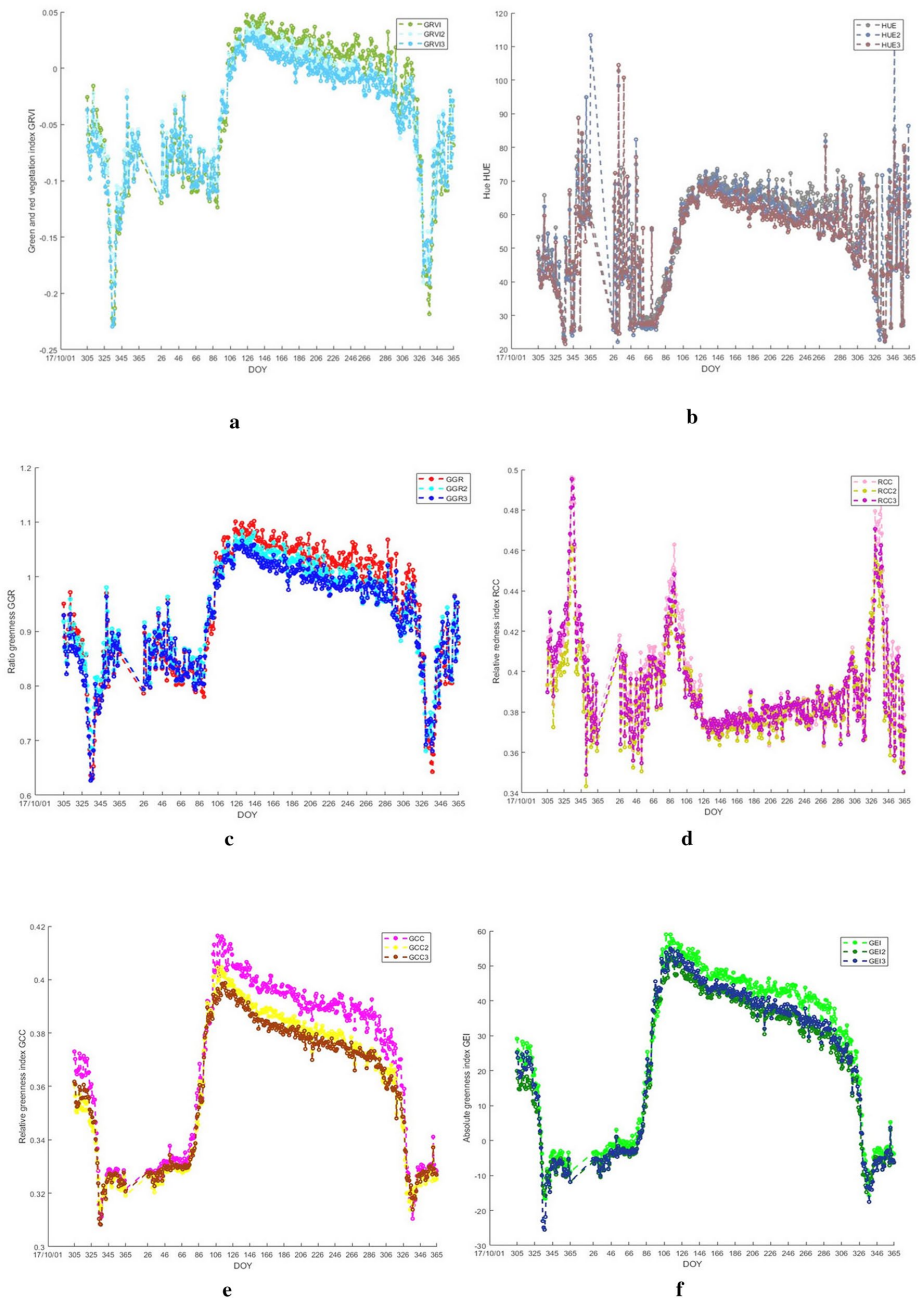
Analysis of the same indicators for the three areas of interest

As shown in Fig. 4, the same GRVI, HUE, GGR, RCC, GCC, and GEI indexes in three different ROIs changed significantly from each other. Data Table 2 further proves that the same color index in different ROIs is slightly different. In terms of GRVI, compared with ROI 2 and 3, ROI 1 is 9 days slower in SOS, 13 days slower in MOE and 4 days more in LOS, and EOS is the same for ROI 1 and ROI 3; as for HUE, SOS and EOS are the same for ROI 1, 2 and 3, while ROI 1 is 13 days slower in MOE and 13 days more in LOS compared with ROI 2 and 3; as for GGR, MOE and EOS are the same for ROI 1, 2 and 3, while ROI is 9 days slower in SOS and 9 days less in LOS compared with ROI 2 and 3; as for RCC, SOS, MOM, and LOS are the same for ROI 1, 2 and 3, while EOS is the same for only ROI 2 and 3; as for GCC and GEI, SOS is the same for ROI 1, 2 and 3, while MOE, LOS and EOS are different among them. This indirectly reflected the differences in the micro-ecological environment caused by different positions in the same forest farm, which was in line with the differences in corruption speed and color of man-made forests.

**Table 2:** Day of year (DOY) in different ROIs

ROI	Index	SOS	MOE	LOS	EOS
1	GRVI	91	145	54	337
2		82	132	50	330
3		82	132	50	337
1	HUE	67	145	78	337
2		67	145	78	337
3		67	132	65	337
1	GGR	91	132	41	337
2		82	132	50	330
3		82	132	50	337
1	RCC	91	145	54	337
2		91	145	54	330
3		91	145	54	330
1	GCC	67	108	41	337
2		67	113	46	334
3		67	117	50	337
1	GEI	67	108	41	337
2		67	113	46	334
3		67	113	46	337

### Comparison of different color indexes in the same ROI

**Fig. 5:**
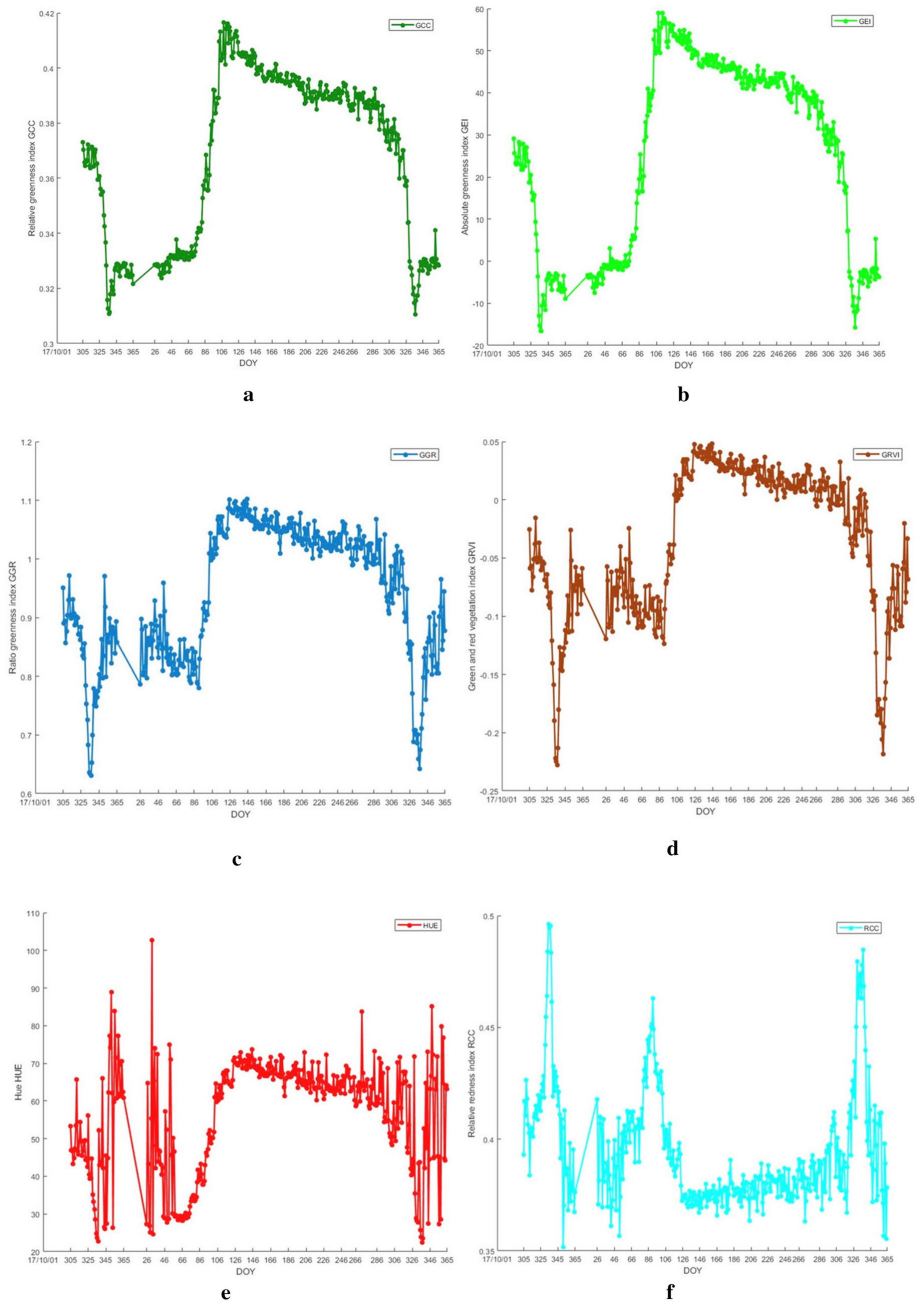
Analysis of different indexes in the same ROI

One pair of data from six color indexes in ROI was selected for extraction and analysis, and the change trends of five green chromatic coordinate indices were consistent, showing a unimodal curve change that increased first, then remained stable, and finally decreased, whereas RCC, in contrast, conformed to the growth phenological changes, but the peak occurrence time was different. The results in Table 3 and Fig. 5 showed that the SOS of GRVI, GGR, RCC and GCC, HUE, and GEI was different; the MOE of GRVI, RCC, and HUE is the largest on Day 145 (May 25), while that of GGR occurs on Day 132 (May 12), and that of GEI and GCC occurs on Day 108 (April 18), which is slightly earlier; in terms of LOS, GGR, GCC, and GEI have different SOS and same LOS, and the LOS of HUE changes greatly; The lowest value data of six color indexes were analyzed, and it was found that their EOS was the same and conformed to the local growth situation. The five-color indexes showed great differences in variation amplitude. In the figure, GCC is consistent with GEI, but the amplitude of GEI is greater than GCC (the variation amplitude can more clearly indicate subtle changes), so GEI is selected for phenological analysis.

**Table 3:** Growth situation in the same ROI

Index	SOS	MOE	LOS (DOY)	EOS
GRVI	91 (April 1)	145 (May 25)	54	337 (December 3)
GGR	91 (April 1)	December 12	41	337 (December 3)
RCC	91 (April 1)	145 (May 25)	54	330 (November 26)
GCC	67 (March 8)	108 (April 18)	41	337 (December 3)
HUE	67 (March 8)	145 (May 25)	78	337 (December 3)
GEI	67 (March 8)	108 (April 18)	41	337 (December 3)

### Time node extraction of vegetation growth characteristics

**Fig. 6:**
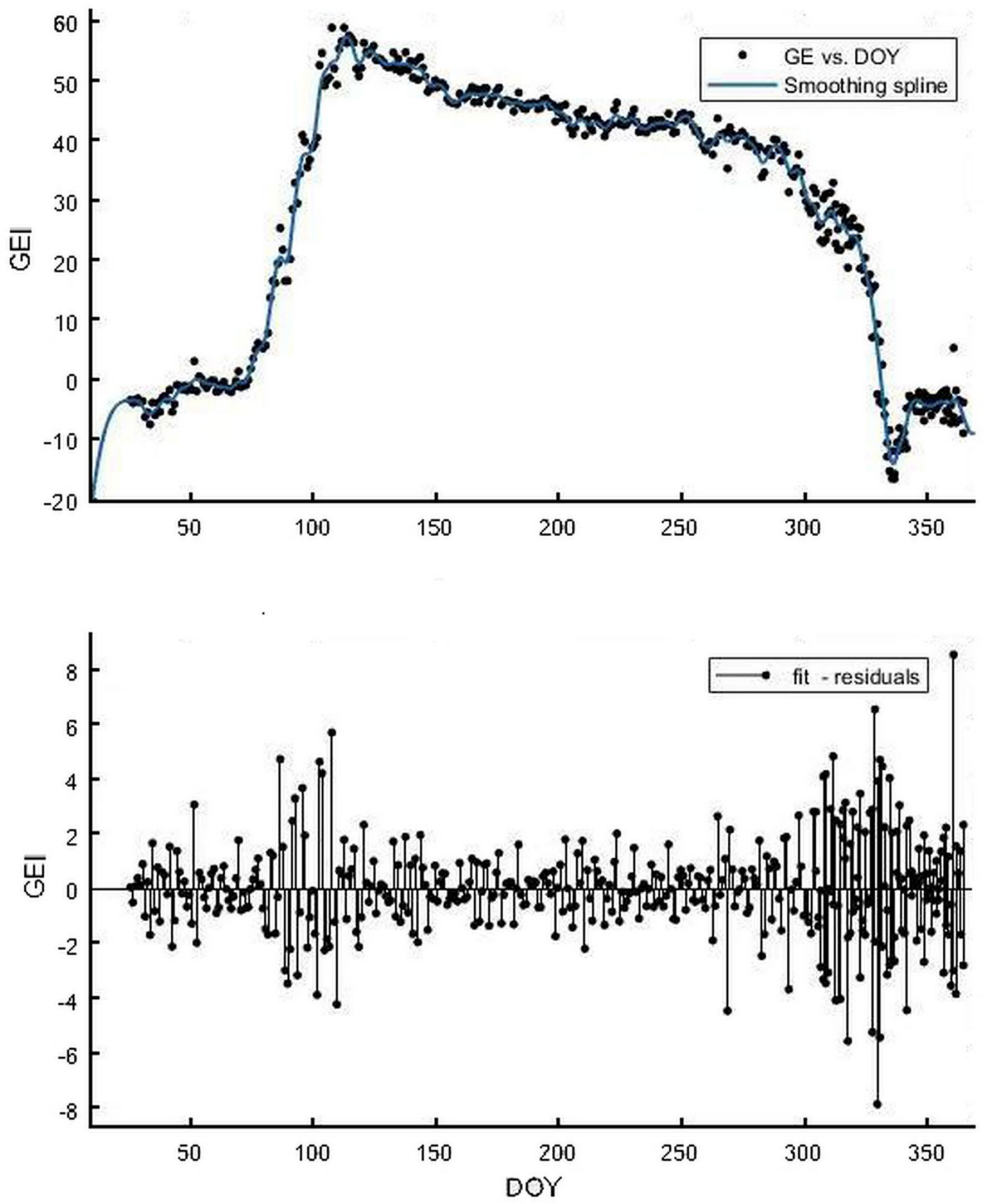
Smooth fitting chart

**Fig. 7:**
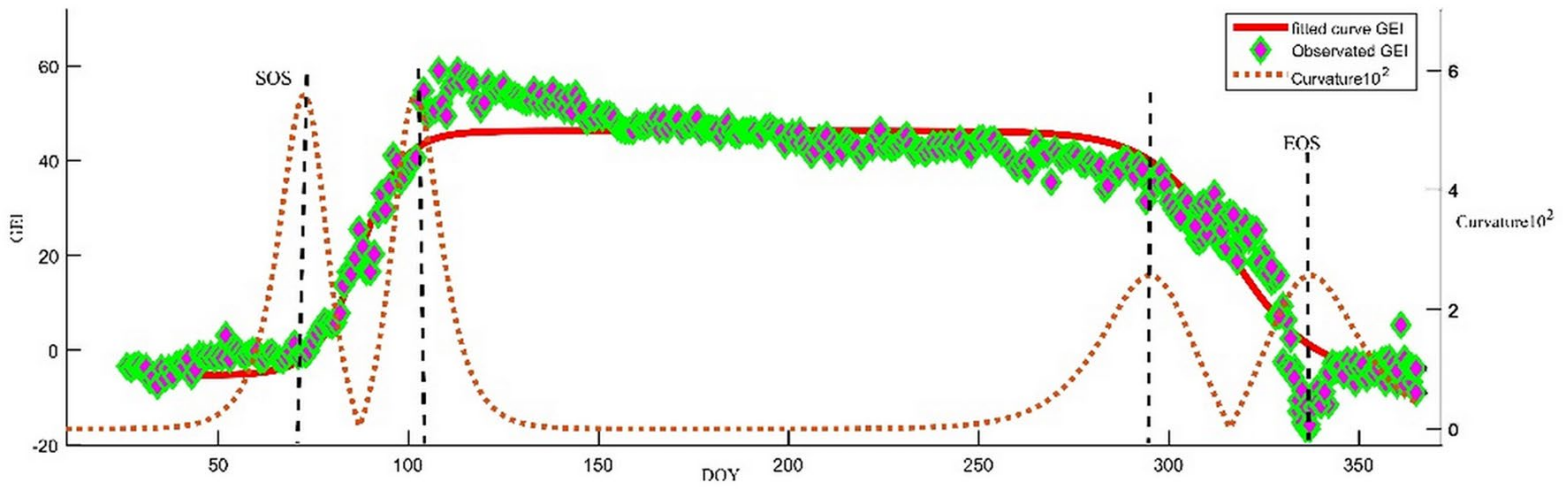
Determination of time sequence and phenological phase of GEI measured value and fitted value

ROI 1 and widely varying GEI color index were selected for phenological analysis. Although affected by daytime differences of environmental factors, smooth fitting (Fig. 6) was required to obtain smoothing parameters: p = 0.032, p < 0.05, fitting residual error RMSE: 2.255, R^^2^ = 0.9912. GEI showed obvious seasonal variation. According to Eqs. (1) and (2), it was concluded that the SOS of the forest was DOY at the beginning when the slope of the curve rises, and similarly, the EOS was DOY at the declining end of the curve (Fig. 7). SOS and the time point of exuberance (Mojzes et al. 2003), COS, and EOS are Day 67, Day 108, Day 288, and Day 337 of the year, respectively. Before Day 67 of the year, the forest was in a period of dormancy, and GEI was affected by snowfall, and the overall change trend was relatively flat; after that, affected by the temperature rise and precipitation increase, the forest gradually germinated, and GEI increased with it, and vegetation SOS began; GEI reached a high value until Day 108 of the year when the forest was exuberant [33], which lasted about 57 days until October 15 (Day 288), when COS began. Since then, forest activities gradually weakened as the temperature declined, and GEI also decreased; on Day 337 of the year, EOS ended and all forests turned yellow, and GEI returned to a low value again.

### Construction of LSTM model

**Fig. 8:**
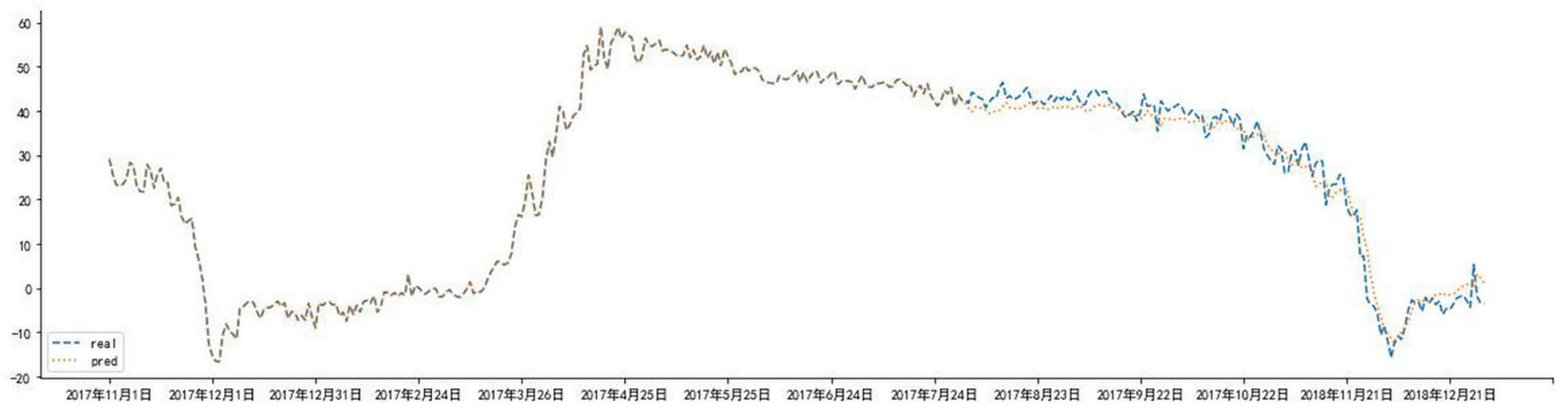
Trend chart of training set and test set

**Fig. 9:**
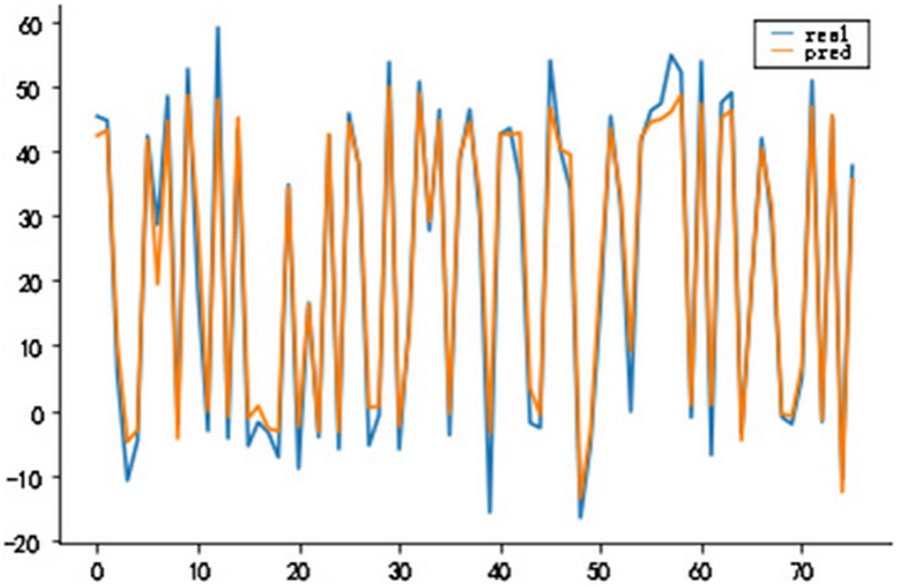
True value and predicted value

**Fig. 10:**
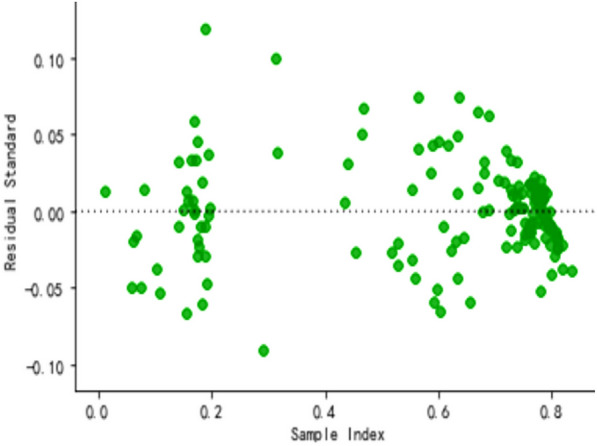
Residual plot of LSTM model

**Fig. 11:**
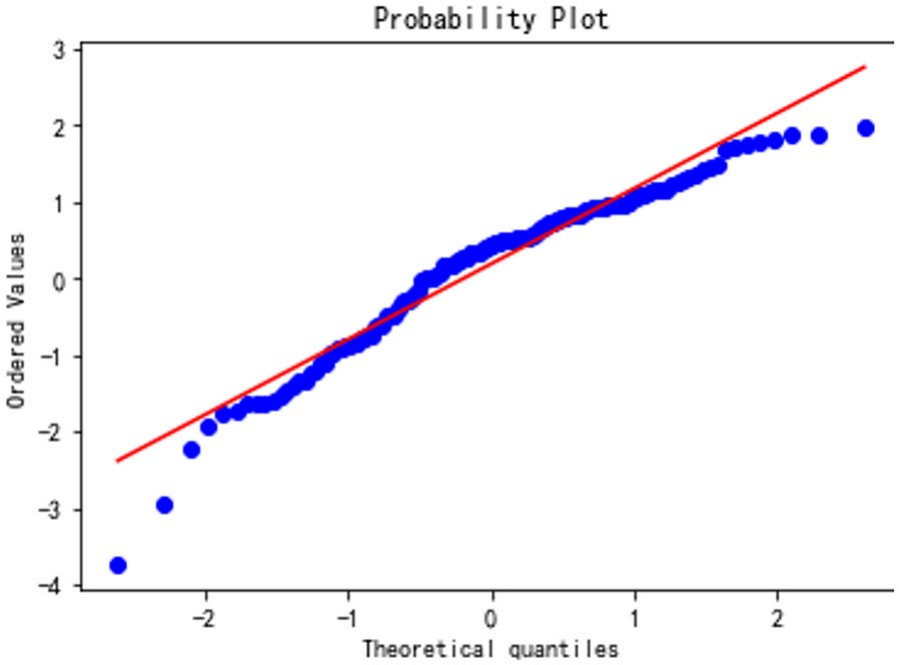
QQ plot testing of LASTM model

In order to observe the forecasting results more intuitively, the actual value and predicted value of the LSTM model were visualized (Fig. 8). The blue dotted line represented the fitting effect of the model on the training set, and the left part represented the forecasting results of the model on the test set. LSTM model showed a trend that the actual value and the predicted value were relatively close (Fig. 9), indicating the feasibility and effectiveness of the GEI color index forecast. MSE, RMSE, MAE, and MAPE were 0.0016, 0.0405, 0.0334 and 12.55%, respectively. It could be found from the results (Figs. 10 and 11) that the model prediction was successful.

**Fig. 12:**
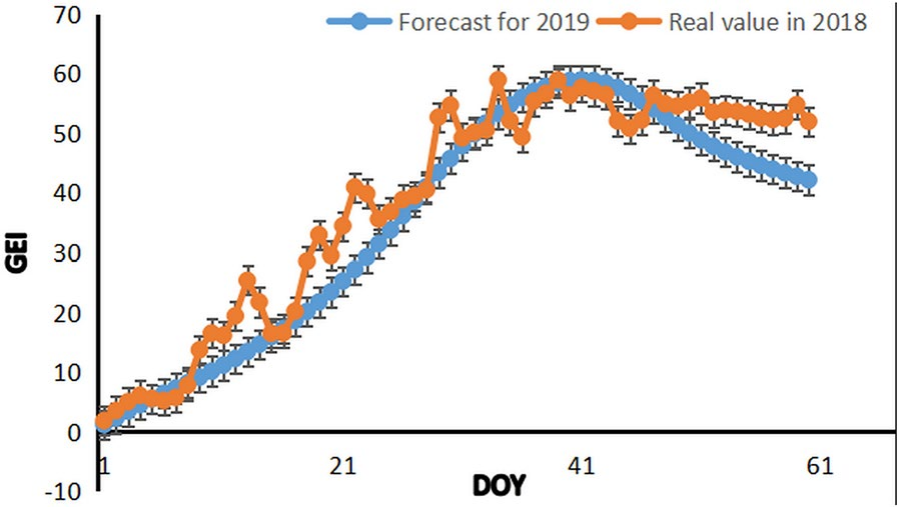
Prognostic chart of color indexes in 2019

The data inside each cell varies with the model window size. The window size represents the amount of past data entered into the model, which is equal to the number of cells in each LSTM cell. As for future forecasts, the window size is set to be 30 days, that is, using 30 days of data as input and the value from Day 31 as the predicted value. According to stepwise training speculation, the color index data on the n_th_ day is subjected to training and learning in sequence, the numerical value at the n + 1st moment is predicted, and the error between the actual value and the predicted value during the training period is minimized to learn the corresponding weight. For the data predicted for the next 60 days, that is, January and February of the second year, the 30-step prediction window is used to input 30 days of data, and the data for the following one day is used as output. The time sequences of input and output remain unchanged. The predicted data is obtained in a Notebook environment to draw the forecast results (Fig. 12). The results showed that the color index of the growth situation in the second half-year gradually rises 60 days ago, which is similar to the growth trend in the first half-year of 2018, indicating that the model achieves a good forecast effect.

## Discussion

This study found that there were slight changes in forest phenological growth studies for different ROIs, and different phenomena reflected by different color indices, and color indexes could be used as phenology forecast indexes, which were consistent with our original hypothesis. To clarify this phenomenon, we extracted the same color index from three different ROIs and six color indexes from the same ROI and trained and predicted the optimal color index based on the LSTM model. The results showed that: (1) the color index of different ROIs has slight differences; (2) extraction and comparison of different color indexes for the same ROI of this forest indicate that GEI is more in line with the phenological growth law; (3) the GEI color index is modeled and predicted based on the LSTM, which shows that the true value and the predicted value approximately tend to be consistent with the model evaluation index, and the predicted future value conform to the change of growth law. The results of our study as a whole showed that the same color index is slightly different in different ROIs, indirectly reflecting the different growth environments in each position, that is, the internal substances in the mechanism such as nutrition in the phenological canopy are different; secondly, compared with the color index in the same ROI, GEI is more in line with the phenological canopy analysis, and can be used as the next forecast index of phenology.

The same color index in different ROIs shows different effects, indicating the existence of a micro-ecological environment in different ROIs, which leads to the difference in chlorophyll production. The distance problem of the selected ROI and the external error caused by the influence of the camera orientation on the image are not excluded. Such difference may be due to unified cultivation or irrigation method, and therefore unbalanced nutrient intake occurs in different ROIs. The six-color indexes in the same ROI could reflect the forest phenological growth phenomenon. By contrast, it is found that both GEI and GCC conform to the forest growth law in this study. GEI is selected in this study after amplitude comparison; previous studies have repeatedly indicated the existence of a microenvironment in forests. For example, the analysis of high-frequency PhenoCam imagery revealed smaller uncertainty than phenology indicators obtained using satellite remote sensing [34]. The near-surface time-series estimates for early spring were found to be in good agreement with estimates derived from the visual assessment of leaf-out and satellite remote sensing data. In deciduous forests, the leaf area index measurements in spring and autumn phenological transition dates are well extracted from digital photography [35]. By examining the aggragate effect of changes in leaf color (green) and canopy structure, it possible to reproduce the observed seasonal trajectory of canopy greenness. For this purpose, a scecial model has been built [35]. Consistent with our research results, ROI at different positions present different color indexes, because the cultivation method and the surrounding environment will affect the growth of forests. It is thus found that the existence of a micro-ecological environment in forests can be reflected with color index observations.

The optimal color index varies with the ecosystem. For example, RCC was more useful than GCC in tracking the canopy photosynthetic phenology of the ENF ecosystem [36]. GCC index was more suitable for monitoring the seasonal variation of Robinia pseudoacacia, but GCC was not sensitive to the seasonal variation of birch [37]; HUE was found to be the most suitable color index from the sensitivity of distinguishing leaf color index in digital images [38], and Sgreen and GEI showed a better correlation with GPP than HUE in rubber plantations [39]; also, it was pointed out that HUE was not related to GPP, and is easily affected by white balance. RCC is considered to be more suitable for estimating the abscission period of evergreen forest [40, 41]; however, it was considered that GEI was more accurate than GCC in characterizing the seasonal variation of grassland [42]. Consistent with our findings, previous studies showed that the six color indexes could reflect the phenological growth period with slight differences, and appropriate color indexes should be selected according to the ecosystem of the tree species studied.

The trend of the true value and the predicted value predicted by the LSTM model was consistent, and the evaluation model indexes showed that the model accuracy was high, and the GEI color index could be used as a phenology forecast index of forest growth in the model prediction. The random forest regression algorithm has been used for the prediction of environmental parameters and relative leaf area index of plant transpiration, as well as the prediction of non-contact and non-destructive chlorophyll content [43]. The yield was predicted according to the vegetation indexes from the regression analysis satellite images [24, 44–46]. In this study, the LSTM model was first applied to forest phenology research, with color indexes as the indicator. The true value and the predicted value showed a good prediction trend, indicating that the error of the predicted value using the LSTM model was small. Throughout the prediction results from the LSTM model, there was a good fitting effect, which conformed to the trend of time sequence data. The prediction in the second half-year also conformed to the trend of growth laws. This study has the following novel points and advantages. For the first time, the extraction and analysis of the same color index extracted from three different ROIs and six color indexes from the same ROI are presented. Secondly, in terms of forest prediction, the color index is used for phenology forecast based on the LSTM model. This indirectly reflects the difference of micro-ecological environment in different positions of the whole forest farm, indicating that the nutrients such as water needed in each position were also different, and different measures should be taken in different positions to achieve scientific cultivation and avoid waste. Our study still has its limitations and lacks internal physiological mechanism research, highlighting the growth and health effects from the surface of the phenological canopy only. The next step will be to study the relationship between the internal physiological mechanism and phenological canopy, as well as the relationship between the change of color indexes and the internal physiological mechanism.

## Conclusions

The same color index has different effects in different ROIs, which indirectly reflects the existence of micro-ecological environment in different positions of the whole forest farm, and then the required nutrient substances such as water are different, so the forest cannot be cultivated in a unified way. It is necessary to implement different measures in different positions, thereby avoiding waste; the six color indexes in the same ROI could reflect the forest phenological growth rule, but different color indexes are slightly different, and the appropriate color index should be selected for phenological analysis of individual species. In particular, this study shows that color index data is used as time sequence data set based on LSTM model, and the data is divided into the training set and the forecast set to obtain good forecast results, and the phenology forecast results in the second half-year conform to the growth trend and data. This study provides a feasible way for studying the relationship between the seasonal variation of vegetation communities and the health factors at different positions and provides an effective means for diagnosing seasonal evolution characteristics of mix artificial forests and diagnosing the rapid response of ecosystems to climate change on the local, regional and global scales.

## Data Availability

Data will be available on request.

## Abbreviations

DRNN: Diagonal recurrent neural network
GCC: The green chromatic coordinate
GEI: The absolute green index
GGR: The specific green index
GRVI: Green–red vegetation index
HUE: The hue
LSTM: Long short term memory
MAE: The mean absolute error
MAPE: The mean absolute percentage error
MSE: Mean squared error
RCC: Red chromatic coordinate
RMSE: Root mean squared error
RNN: Recurrent neural network
ROI: Region of interest
